# Supplementary material for: Pre-existing immunity and vaccine history determine hemagglutinin-specific CD4 T cell and IgG response following seasonal influenza vaccination
Source: Nat Commun. 2021 Nov 18;12:6720. doi: 10.1038/s41467-021-27064-3 (PMC8602312; doi:10.1038/s41467-021-27064-3)
Supplement: Supplementary file 1 — Supplementary Information [file 41467_2021_27064_MOESM1_ESM.pdf]

Supplementary Fig. 1

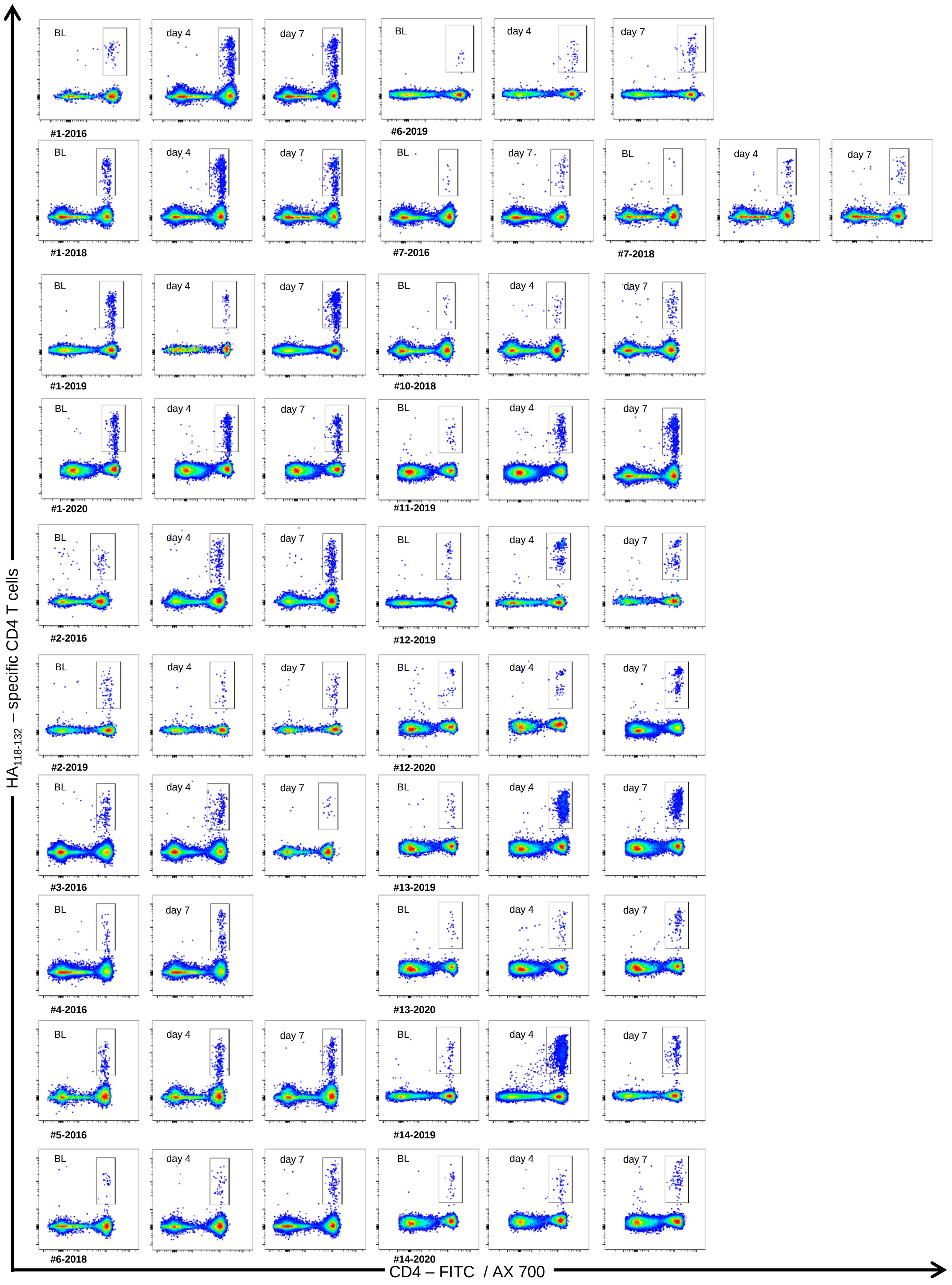

Supplementary Fig. 1: Pseudocolour plots of $HA_{118\text{-}132}$ -specific CD4 T cells of all responses on BL, day 4 and day 7.

Supplementary Fig. 2

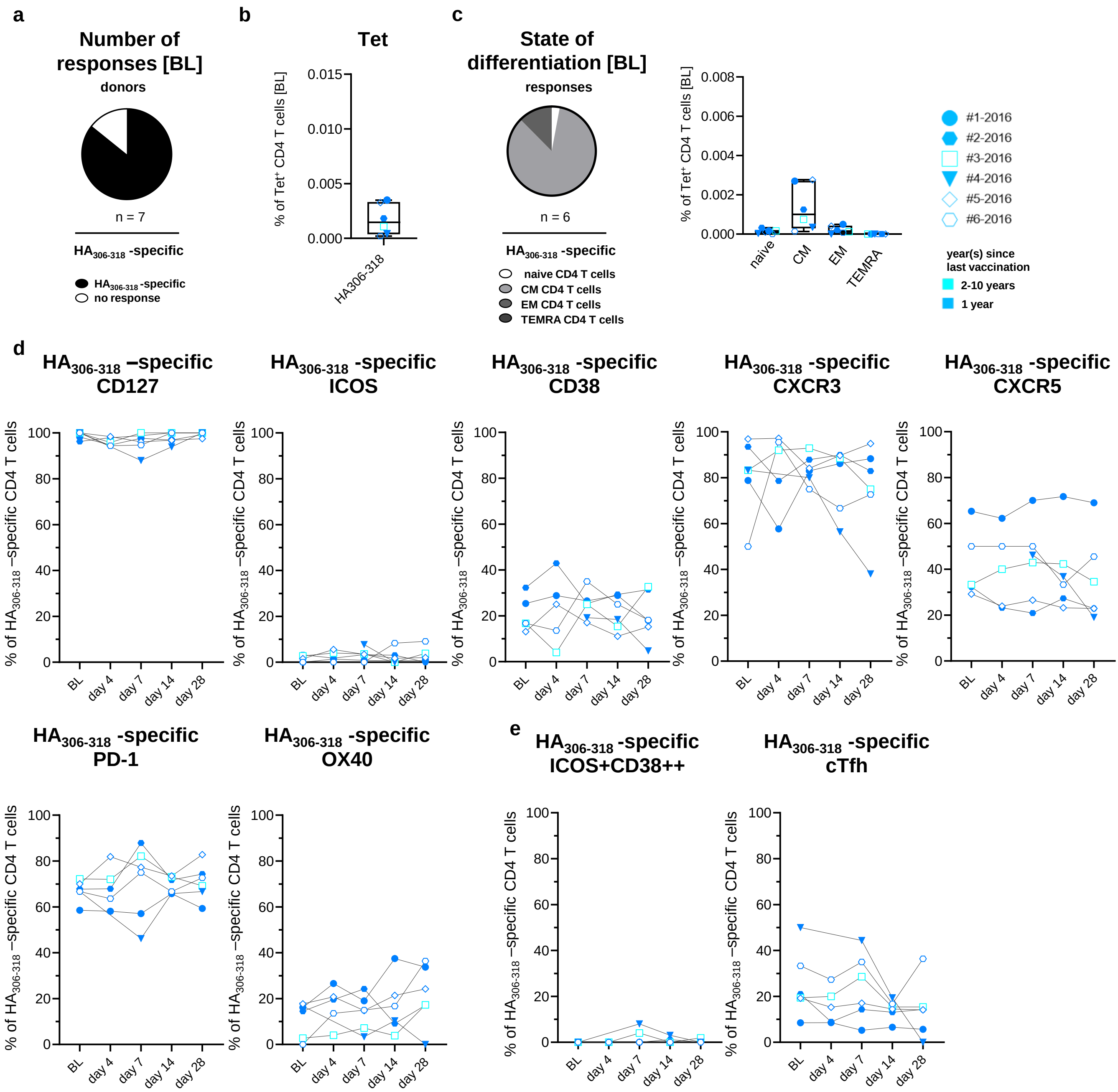

Supplementary Fig. 2: Longitudinal phenotypic analysis of HA$_{306-318}$ -specific CD4 T cells. a Number of detected responses with HA$_{306-318}$ -specific tetramers at BL (n = 7). **b** Frequency of HA$_{306-318}$ -specific cells within CD4 T cells at BL. **c** State of memory differentiation of HA$_{306-318}$ -specific CD4 T cells at BL. **d** Indicated marker expression on HA$_{306-318}$ -specific CD4 T cells at the indicated time points. **e** Frequency of ICOS+CD38++ and cTfh HA$_{306-318}$-specific CD4 T cells at the indicated time points. One symbol represents one response at the indicated time point. Responses are sub grouped by vaccination history. (HA$_{306-318}$-specific CD4 T cell responses for BL, day 7 – day 28 n = 6, day 4 n = 5). Two-Tailed Wilcoxon matched-pairs signed rank test with Bonferroni correction for multiple comparisons was used for between group comparisons. All time points were compared to BL. In **(d-e)** p of 0.0125 was considered as statistically significant.

Supplementary Fig. 3

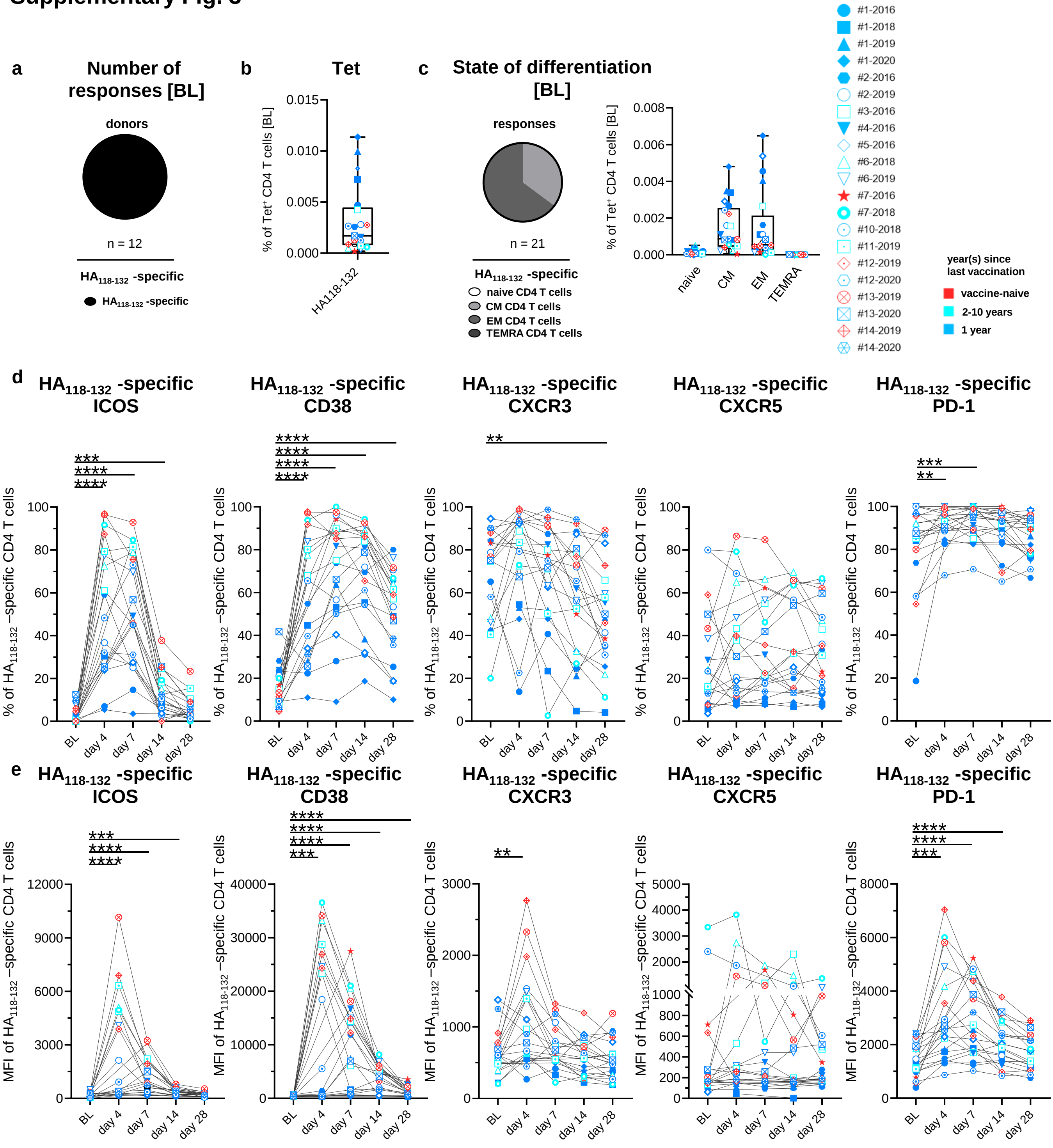

Supplementary Fig. 3: Longitudinal phenotypic analysis of $HA_{118-132}$ -specific CD4 T cells. a Number of detected responses with $HA_{118-132}$-specific tetramers at BL (n = 12). **b** Frequency of $HA_{118-132}$-specific cells within CD4 T cells at BL. **c** State of memory differentiation of $HA_{118-132}$-specific CD4 T cells at BL. **d** and **e** show longitudinal analysis of markers included in the heatmap Fig 2d. **d** Frequency of marker-positive $HA_{306-318}$ -specific CD4 T cells at the indicated time points. **e** MFI of marker-positive $HA_{306-318}$ -specific CD4 T cells at the indicated time points. One symbol represents one response at the indicated time point. Responses are sub grouped by vaccination history (red n = 4, turquoise n = 4, blue = 13). Two-tailed Wilcoxon matched-pairs signed rank test with Bonferroni correction for multiple comparisons was used for between group comparisons. All time points were compared to BL. In **(d-e)** p of 0.0125 was considered as statistically significant. ** $p < 0.01$, *** $p < 0.001$, **** $p < 0.0001$.

Supplementary Fig. 4

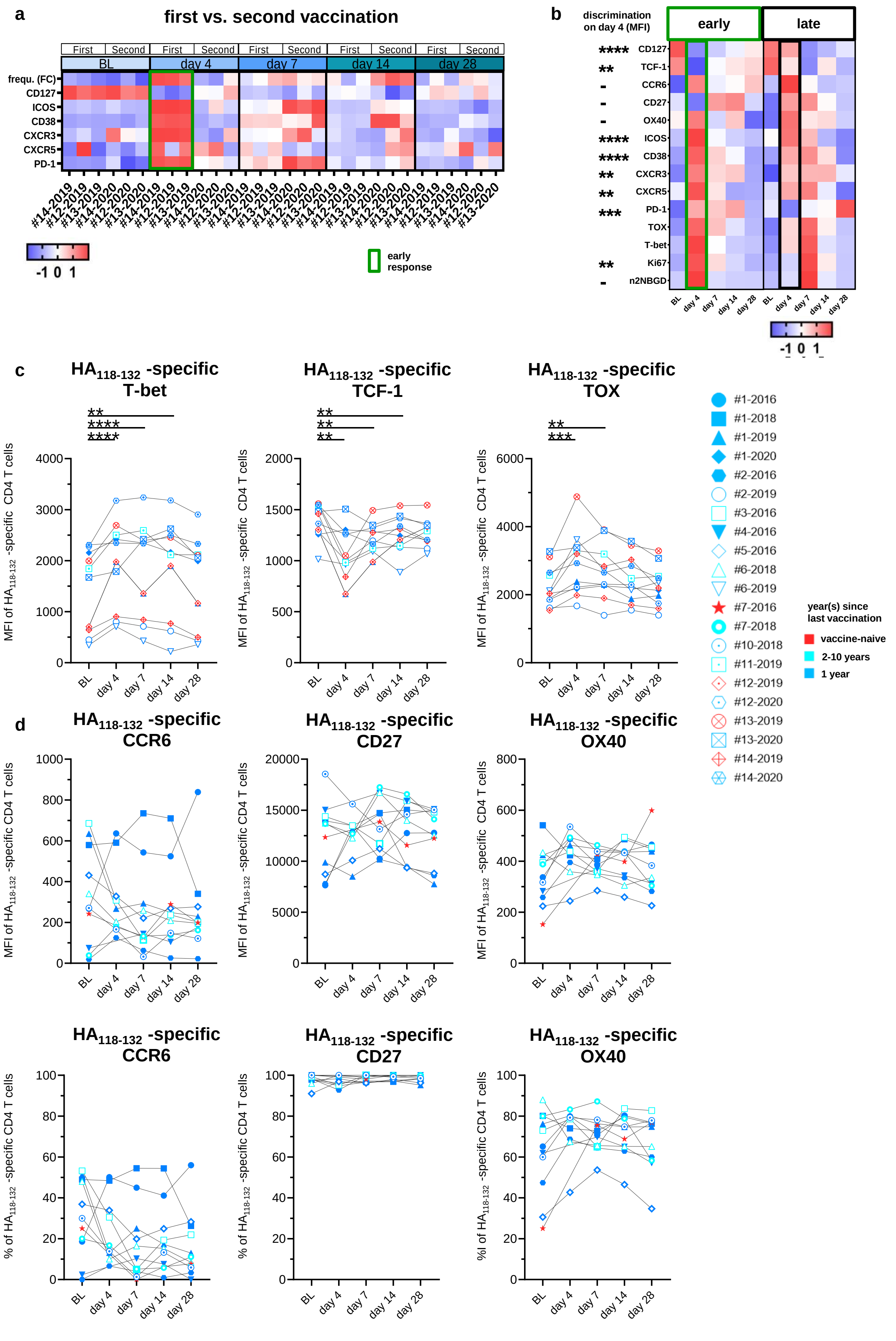

Supplementary Fig. 4: Summarized longitudinal phenotypic analysis of $HA_{118\text{-}132}$ -specific CD4 T cells divided by the different activation patterns. a Separate view of 6 individual responses (extracted from Fig. **2e**) for 3 donors receiving their first and second vaccination. Heatmap colouring represents the z score of every marker (MFI) calculated among the time points of an individual donor response. **b** Summarized heatmap of all analysed parameters in all early (green n = 9) and late (black n = 10) responses as z score (per marker) of the mean fold change (FC, mean from all responses among one time point). FC was calculated per donor for every time point compared to BL. Median fluorescence intensity (MFI) values were used for statistical comparison between early and late responses day 4. Line = no statistical analysis due to low number of early responses. **c** Longitudinal analysis of relevant transcription factors, MFI (red n = 3, turquoise n = 1, blue = 7). **d** Longitudinal analysis of additional surface markers included in summarized heatmap **(b)** (top: MFI, down: %; (red n = 4, turquoise n = 4, blue = 13)). One symbol represents one response at the indicated time point. Two-tailed Wilcoxon matched-pairs signed rank test with Bonferroni correction for multiple comparisons was used for between group comparisons. In **(c, d)** all time points were compared to BL with p of 0.0125 as statistically significant, * $p < 0.0125$, ** $p < 0.01$, *** $p < 0.001$, **** $p < 0.0001$.

Supplementary Fig. 5

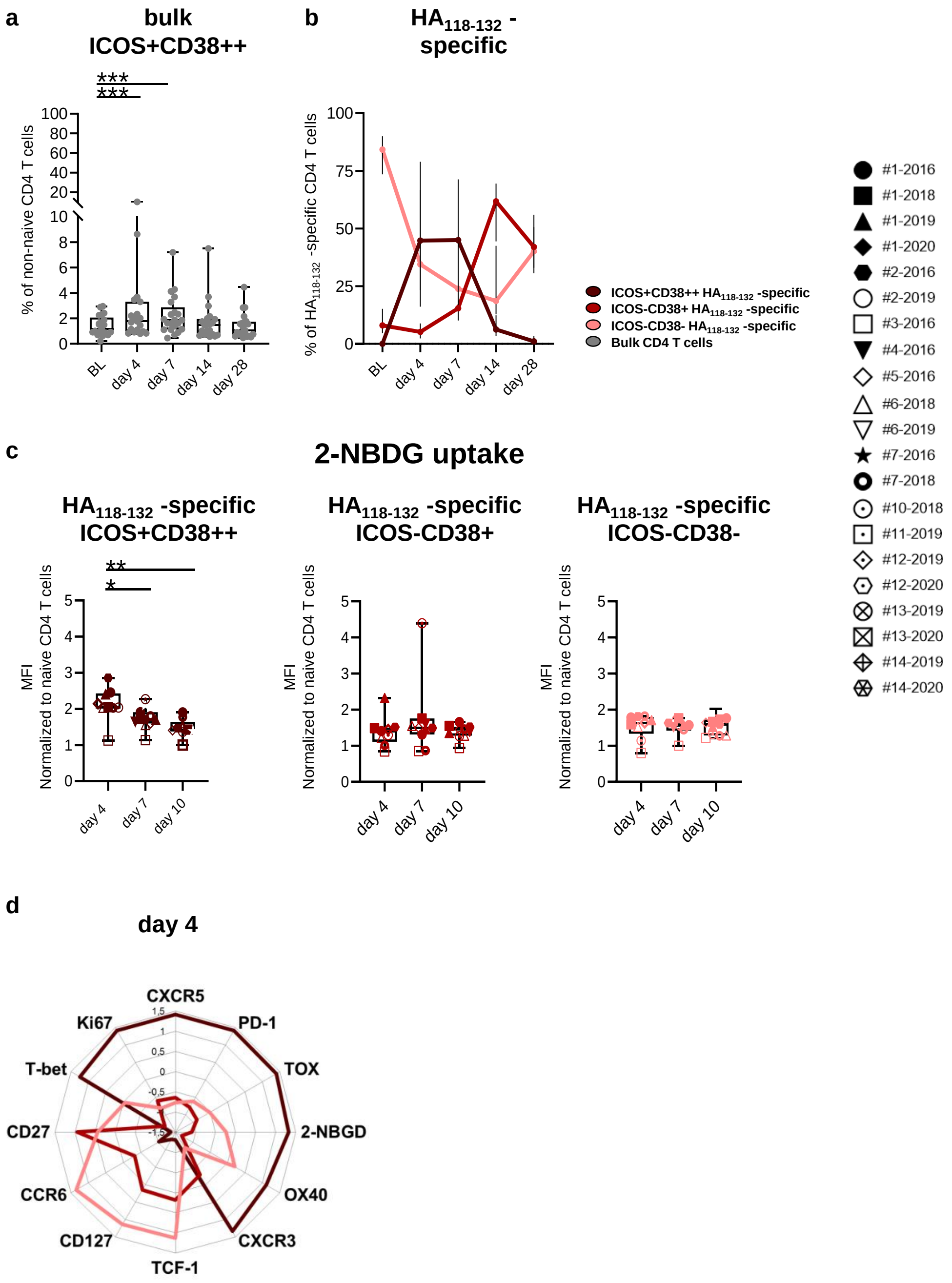

Supplementary Fig. 5: Comparative analysis of differentially activated populations within $HA_{118-132}$ -specific CD4 T cells. a Frequency of ICOS+CD38++ non-naïve CD4 T cells (bulk) at the indicated time points (n = 21). **b** Analysis of 3 populations defined by CD38 and ICOS expression within $HA_{118-132}$ -specific CD4 T cells (n = 21). Every symbol represents the median of analysed responses. Error bars show the 95 % confidence interval. **c** Normalized MFI of 2-NBGD-positive $HA_{118-132}$ -specific CD4 T cells within the different activated populations at the indicated time points. One symbol represents one response at the indicated time points. (day 4 n = 9; day 7 n = 11, day 10 n = 9). **d** Spider plot showing the summarized phenotype of the differentially activated population on day 4. Z scores of the mean marker expressions between the 3 populations are shown. Two-tailed Wilcoxon matched-pairs signed rank test with Bonferroni correction for multiple comparisons was used for between group comparisons. In **(a)** all time points were compared to BL, whereas in **(c)** all populations were compared to day 4. In **(a)** p of 0.0125 (*) an in **(c)** p of 0.025 (*) were considered as statistically significant. ** $p < 0.01$, *** $p < 0.001$.

Supplementary Fig. 6

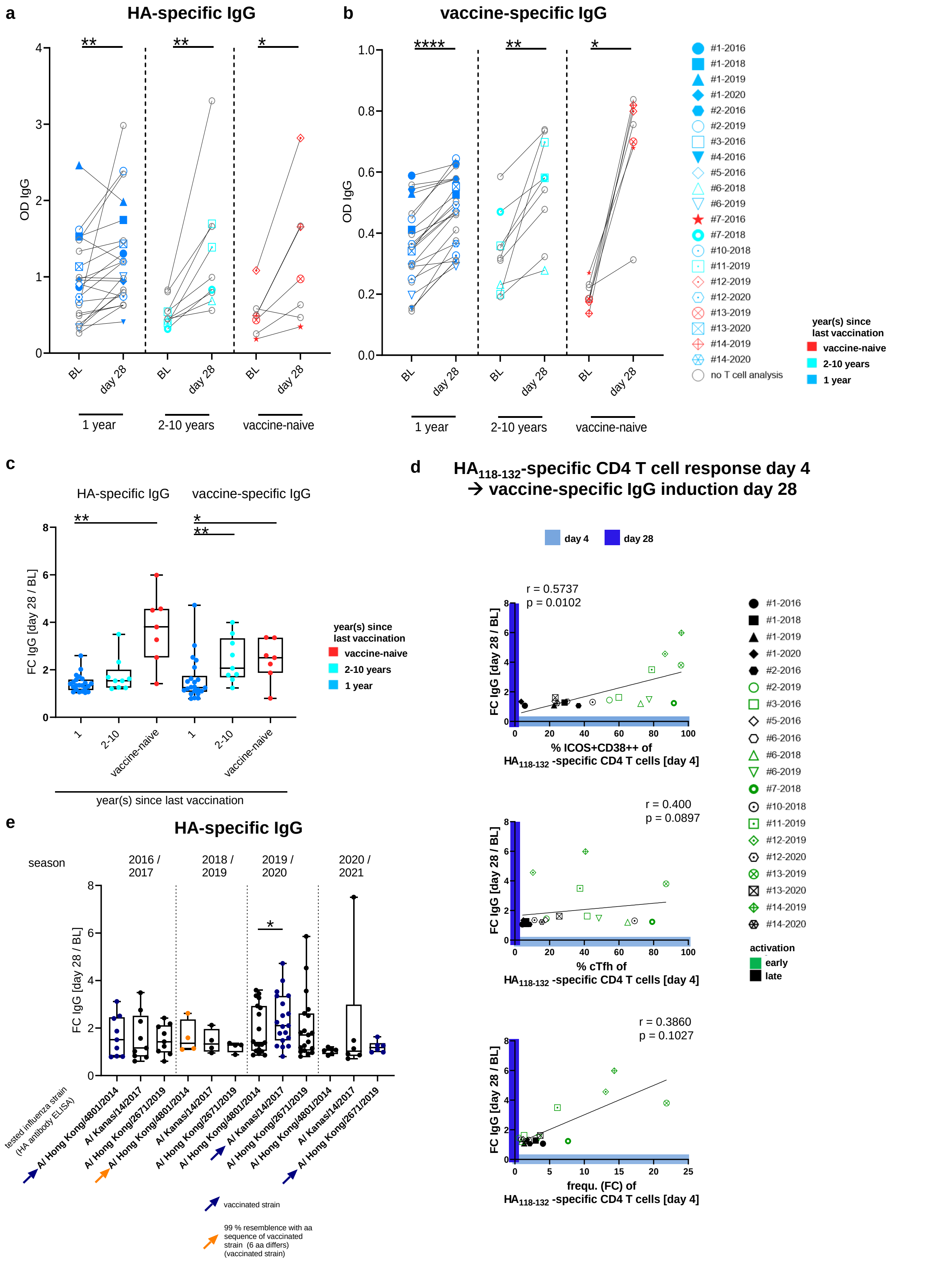

Supplementary Fig. 6: HA- and vaccine-specific antibody analysis. HA-specific IgG plasma levels (**a)** in comparison to vaccine-specific IgG plasma levels **(b)**. Grey circles additionally show antibody responses with no corresponding T cell analysis (due to HLA type) (n = 38). **c** Comparison of HA-specific and vaccine-specific IgG induction (FC day 28 / BL) (n = 38). Plasma levels are sub grouped by vaccination history (red: n = 4, turquoise n = 4, blue = 13). **d** Spearman correlation of ICOS, CD38 co-expressing $HA_{118-132}$ -specific CD4 T cells at day 4, CXCR5 and PD-1 co-expressing $HA_{118-132}$ -specific CD4 T cells at day 4 and frequ. (FC) of $HA_{118-132}$ -specific CD4 T cells [day 4 / BL] with induction of vaccine-specific IgG [day 28 / BL] (n = 19). Responses are sub grouped by early and late responders (green n = 9, black n = 10). **e** Comparison of the HA-specific IgG induction (FC day 28 / BL) measured with different virus strains. The blue arrow indicates the vaccine strain used in the respective year. The orange arrow indicates 99 % similarity with the amino acid sequence of the vaccinated strain, that was used for analysis in the respective years to replace a missing strain. Bars represent the median. **(a, b)** Two-tailed Wilcoxon matched-pairs signed rank test were used for between group comparisons. * $p < 0.05$, ** $p < 0.01$, *** $p < 0.001$, **** $p < 0.0001$. **(c, e)** Two-tailed Mann-Whitney U-test was used with Bonferroni correction for comparisons between two groups. p values of 0.025 was considered as statistically significant. * $p < 0.025$, ** $p < 0.01$ **(d)** Two-tailed spearman correlation test with a confidence interval of 95 %. * $p < 0.05$. One symbol represents one response at the indicated time point.

Supplementary Fig. 7

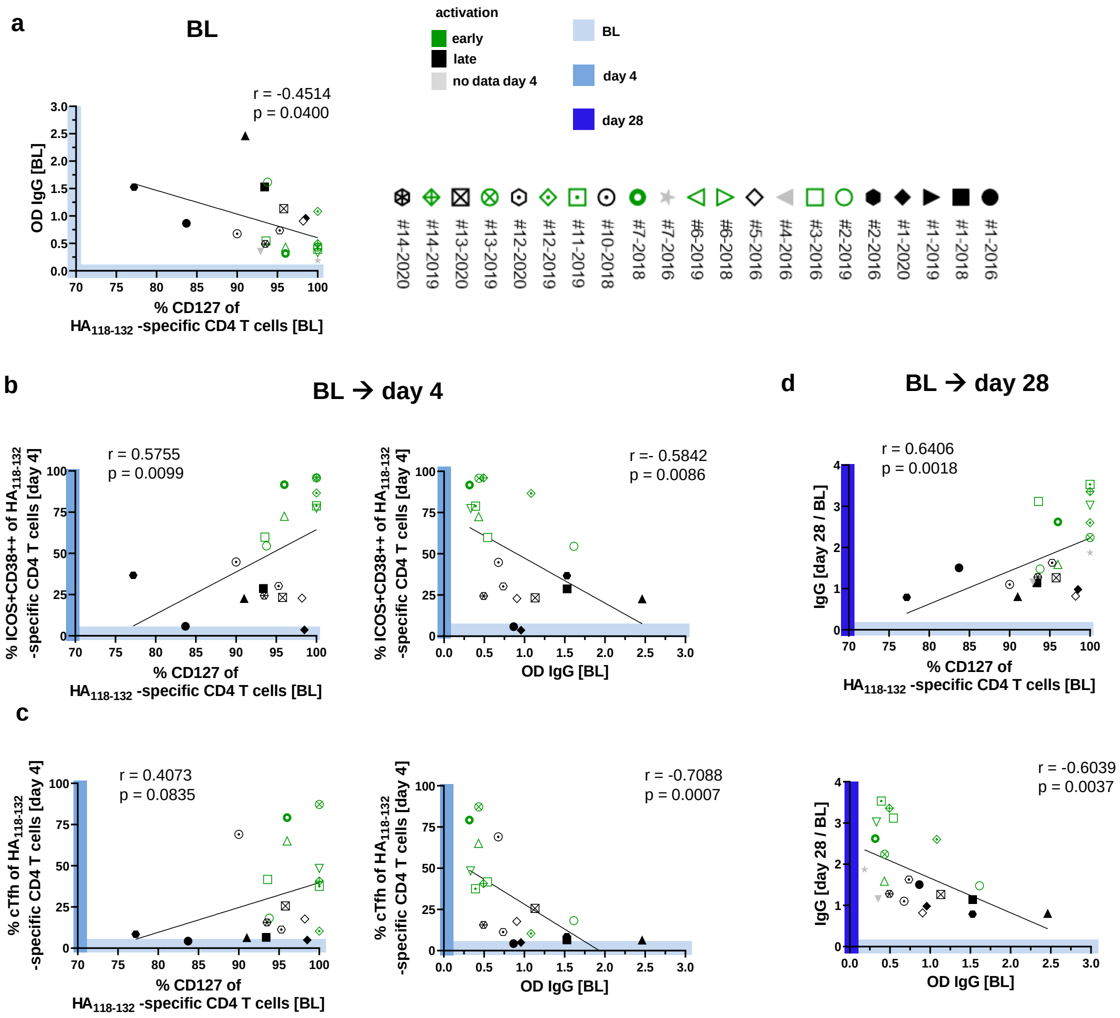

Supplementary Fig. 7: The most relevant correlations from Fig. 6c with early and late $HA_{118-132}$-specific CD4 T cell responses marked in the graphs. a Spearman correlation of CD127 expressing $HA_{118-132}$-specific CD4 T cells [BL] with HA-specific IgG levels [BL] (n = 19). **b-c** Relevant spearman correlations of BL parameters with parameters day 4 (n = 19). **d** Relevant spearman correlations of BL parameters with HA-specific antibody induction (FC BL / day 28) (n = 19). Two-tailed spearman correlation test with a confidence interval of 95 %. One symbol represents one response at the indicated time point. * $p < 0.05$, ** $p < 0.01$, *** $p < 0.001$.

Supplementary Fig. 8

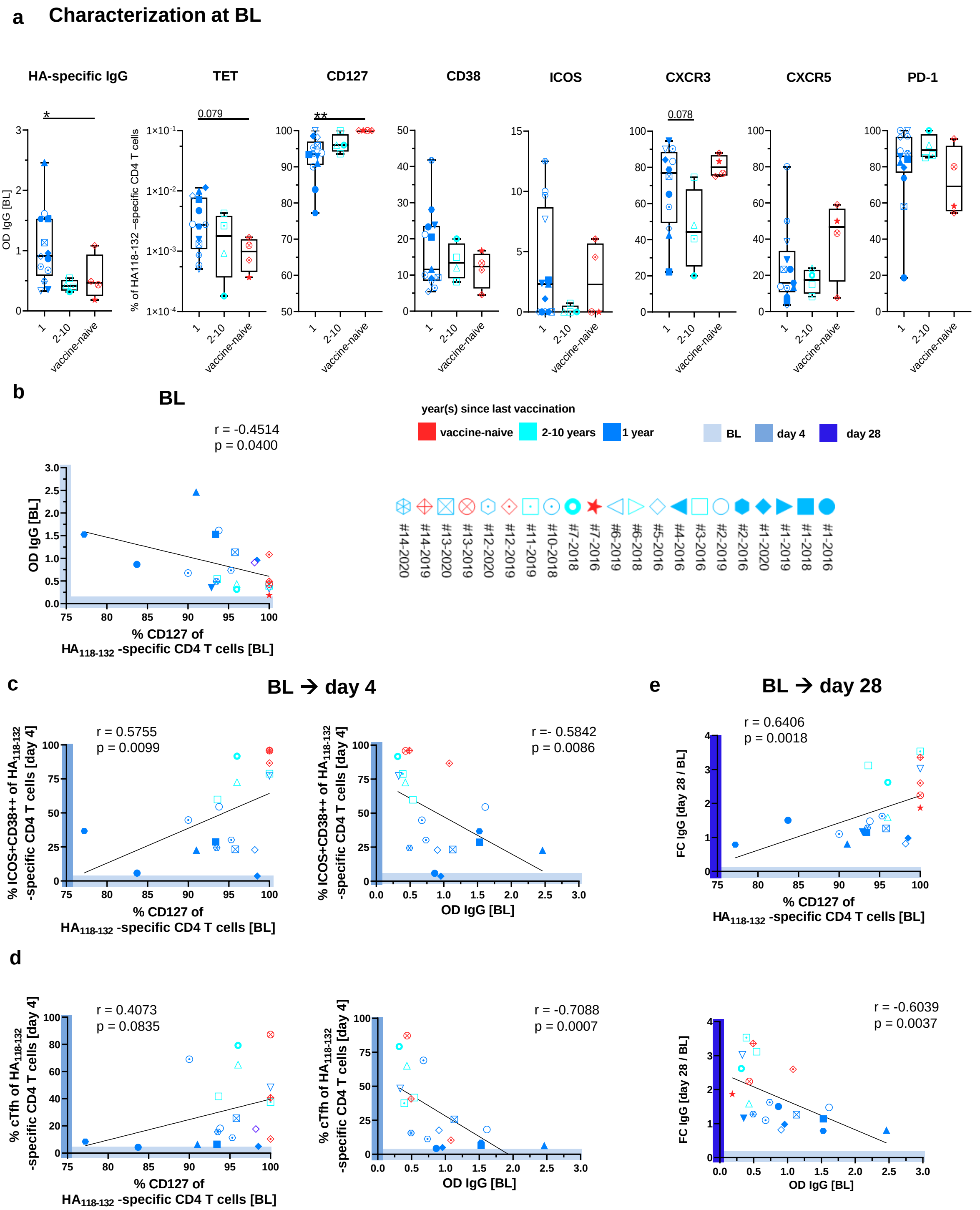

Supplementary Fig. 8: BL parameters that influence the dynamics of $HA_{118-132}$-specific CD4 T cell activation and HA-specific IgG induction after seasonal vaccination. This figure shows data from Fig. 6 sub grouped by vaccination history and the most relevant correlations. **a** Analysis of BL parameters to discriminate responses by vaccination history. Two-tailed Mann-Whitney U-test was used with Bonferroni correction for comparisons between two groups. A p values of 0.025 was considered as statistically significant. * $p < 0.025$, ** $p < 0.01$, (red n = 4, turquoise n = 4, blue = 13). **b** Spearman correlation of % CD127+ $HA_{118-132}$-specific CD4 T cells [BL] with HA-specific IgG levels [BL] (n = 19). **c, d** Relevant spearman correlations of BL parameters with parameters day 4 (n = 19). **e** Relevant spearman correlations of BL parameters with HA-specific antibody induction (FC BL / day 28) (n = 19). **(b – e)** Two-tailed spearman correlation test with a confidence interval of 95 %. One symbol represents one response at the indicated time point. * $p < 0.05$, ** $p < 0.01$, *** $p < 0.001$.

Supplementary Fig. 9

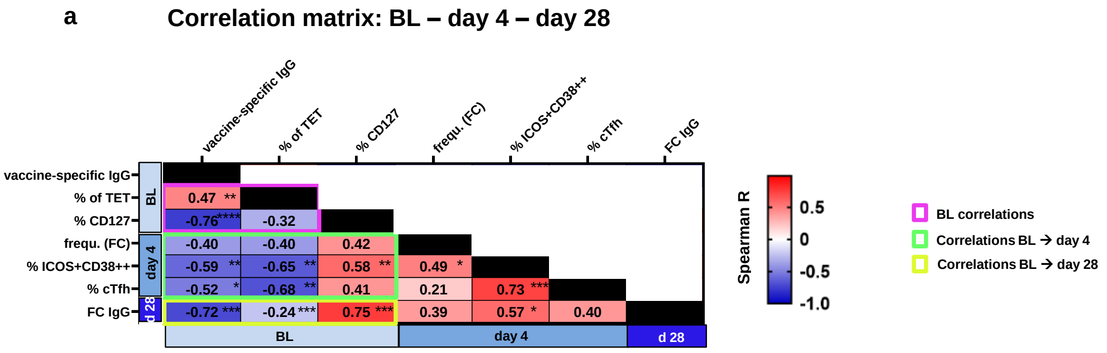

Supplementary Fig. 9: Correlation matrix with data of vaccine-specific IgGs. Correlation matrix with spearman r value from BL to day 4 and day 28 (n = 19 responses). Two-tailed spearman correlation test with a confidence interval of 95 %. * p < 0.05, ** p < 0.01, *** p < 0.001, **** p < 0.0001.

Supplementary Fig. 10

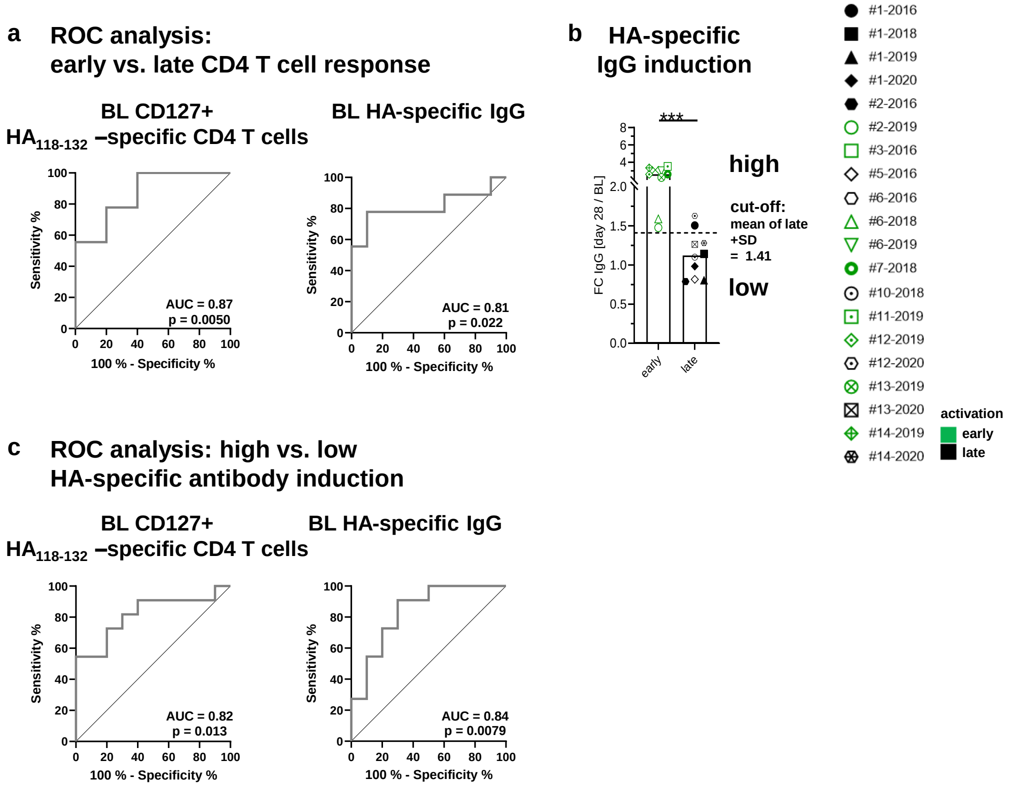

Supplementary Fig. 10: Predictive capacity of BL parameters. a ROC analysis of BL parameters for discrimination of early and late $HA_{118-132}$ -specific CD4 T cell responses. **b** HA-specific IgG induction (FC day 28 / BL) during early and late responses. The cut-off value separates high vs. low antibody induction (green n = 9, black: n = 10). **c** ROC analysis of BL parameters for discrimination of high vs. low antibody induction. **(b)** Two-tailed Mann-Whitney U-test was used for comparisons between two groups. One symbol represents one response at the indicated time point. * p < 0.05, ** p < 0.01, *** p < 0.001. **(a, c)** ROC analysis was performed with 95 % confidence interval and Wilson / Brown method. * p < 0.05, ** p < 0.01. ROC: receiver operating characteristic.

Supplementary Fig. 11

a **Gating strategy: Flow cytometry**

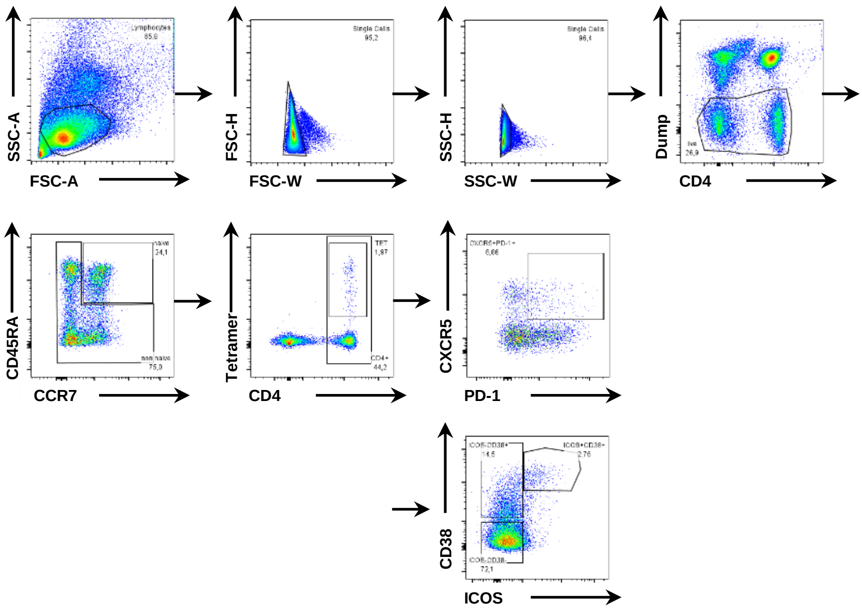

b **Gating strategy: tSNE analyis**

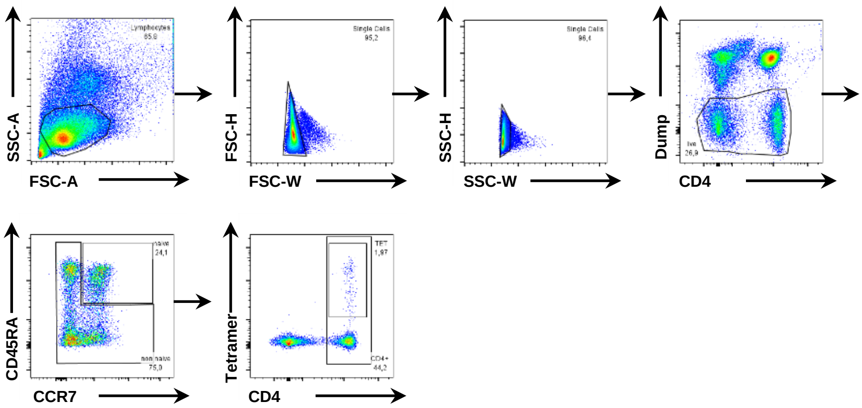

Supplementary Fig. 11: Gating strategy to identify and analyze influenza-specific CD4 T cells. **a** Flow cytometry analysis **b** tSNE analysis.

.

Supplementary Table 1: Used Tetramers (MBL, Woburn, USA)

Tetramer	HLA class II molecule	Influenza Virus A	peptide epitope sequence	amino acid location
HA306-318	DRB1*01:01	H3N2	PKYVKQNTLKLAT	306-318
HA118-132	DRB1*01:01	H3N2	VPDYASLRSLVASSG	118-132

Supplementary Table 2: Antibodies and fluorescent dyes						
Antigen	Fluorophore	Clone	Supplier	Staining Dilution	Staining	Panel
CD4	FITC	RPA-T4	BD Biosciences	1:50	extracellular	Panel 1
CD4	AlexaFluor700	RPA-T4	BioLegend	1:200	extracellular	Panel 2
CD45RA	PerCP-Cy5.5	HI100	Invitrogen	3:100	extracellular	Panel 1 / Panel 2
CD14	eFluor780	61D3	eBioscience	1:100	extracellular	Panel 1 / Panel 2
CD19	eFluor780	HIB19	eBioscience	1:100	extracellular	Panel 1 / Panel 2
ViaDye	eFluor780		eBioscience	1:200	extracellular	Panel 1/ Panel 2
CCR7	BUV395	3D12	BD Biosciences	4:100	extracellular	Panel 1
CXCR5	APC	RF8B2	BD Biosciences	1:50	extracellular	Panel 1
CXCR5	BV421	J252D4	BioLegend	1:100	extracellular	Panel 2
CXCR3	BV510	G025H7	BioLegend	3:100	extracellular	Panel 1 / Panel 2
PD-1	BV786	EH12.1	BD Biosciences	3:100	extracellular	Panel 1 / Panel 2
CD134 (OX40)	PE-Cy7	ACT-35	BD Biosciences	3:100	extracellular	Panel 1
CD38	BUV737	HB7	BD Biosciences	1:200	extracellular	Panel 1/ Panel 2/
ICOS	BV711	DX29	BD Biosciences	1:100	extracellular	Panel 1 / Panel 2
CD127	BV421	HIL-7R-M21	BD Biosciences	3:100	extracellular	Panel 1
CD127	BV605	A019D5	BioLegend	3:100	extracellular	Panel 2
CCR6	BV605	11A9	BD Biosciences	3:100	extracellular	Panel 1
CD27	PE-Dazzle594	M-T271	BioLegend	1:400	extracellular	Panel 1
	2-NBDG (20mM)		Invitrogen	1:200	extracellular	Panel 1
TCF-1	AlexaFluor488	C63D9	Cell signaling	1:100	intranuclear	Panel 2
T-bet	PE-CF594	O4-46	BD Biosciences	3:100	intranuclear	Panel 2
Ki67	PE-Cy7	Ki67	BioLegend	1:200	intranuclear	Panel 2
TOX	eFluor660	TRX10	eBioscience	1:100	intranuclear	Panel 2
