# Supplementary material for: Pre-existing immunity and vaccine history determine hemagglutinin-specific CD4 T cell and IgG response following seasonal influenza vaccination
Source: Nat Commun. 2021 Nov 18;12:6720. doi: 10.1038/s41467-021-27064-3 (PMC8602312; doi:10.1038/s41467-021-27064-3)
Supplement: Supplementary file 3 — Reporting Summary [file 41467_2021_27064_MOESM3_ESM.pdf]

nature research

Corresponding author(s): Tobias Boettler

Last updated by author(s): Oct 4, 2021

Reporting Summary

Nature Research wishes to improve the reproducibility of the work that we publish. This form provides structure for consistency and transparency in reporting. For further information on Nature Research policies, see our Editorial Policies and the Editorial Policy Checklist.

Statistics

For all statistical analyses, confirm that the following items are present in the figure legend, table legend, main text, or Methods section.

n/a	Confirmed	
☐	☒	The exact sample size (*n*) for each experimental group/condition, given as a discrete number and unit of measurement
☐	☒	A statement on whether measurements were taken from distinct samples or whether the same sample was measured repeatedly
☐	☒	The statistical test(s) used AND whether they are one- or two-sided<br>*Only common tests should be described solely by name; describe more complex techniques in the Methods section.*
☐	☒	A description of all covariates tested
☐	☒	A description of any assumptions or corrections, such as tests of normality and adjustment for multiple comparisons
☐	☒	A full description of the statistical parameters including central tendency (e.g. means) or other basic estimates (e.g. regression coefficient) AND variation (e.g. standard deviation) or associated estimates of uncertainty (e.g. confidence intervals)
☐	☒	For null hypothesis testing, the test statistic (e.g. *F*, *t*, *r*) with confidence intervals, effect sizes, degrees of freedom and *P* value noted<br>*Give P values as exact values whenever suitable.*
☒	☐	For Bayesian analysis, information on the choice of priors and Markov chain Monte Carlo settings
☐	☒	For hierarchical and complex designs, identification of the appropriate level for tests and full reporting of outcomes
☐	☒	Estimates of effect sizes (e.g. Cohen's *d*, Pearson's *r*), indicating how they were calculated

Our web collection on statistics for biologists contains articles on many of the points above.

Software and code

Policy information about availability of computer code

Data collection	All software used to perform data collection are described in the methods section of the manuscript. Multiparametric Flow cytometry data was collected by FACSDiva Software (Becton Dickinson). Vaccine-specific IgG ELISA data were acquired and calculated using SparkControl magellan software 2.2. HA-specific IgG ELISA data were acquired using BioTek Plate Reader(Winooski, VT, USA).
Data analysis	All codes and software versions used to perform bioinformatic analyses are described in the methods section of the manuscript or provided in in a github repository.<br>Multiparametric Flow cytometry data was analyzed using FlowJo software version 10.0.7 (LLC, BD Life Sciences, Ashland, OR, USA).<br>Heatmap analysis with unbiased clustering was done with R version 3.6.3 using the ComplexHeatmap package (Gu ZG, Eils R, Schlesner M (2016)). R code to reproduce the analyses of multiparametric flow-cytometry data is available online: https://github.com/teximmed2-fr/flu-specific-CD4-T-cells.<br>A workflow including dimension reduction using tSNE and FlowSOM clustering analysis was implemented in Omiq (Omiq, Inc.). Visualization and statistical analysis was performed using GraphPad 8 software.

For manuscripts utilizing custom algorithms or software that are central to the research but not yet described in published literature, software must be made available to editors and reviewers. We strongly encourage code deposition in a community repository (e.g. GitHub). See the Nature Research guidelines for submitting code & software for further information.

Data

Policy information about availability of data

All manuscripts must include a data availability statement. This statement should provide the following information, where applicable:

- Accession codes, unique identifiers, or web links for publicly available datasets
- A list of figures that have associated raw data
- A description of any restrictions on data availability

Raw data in this study are provided in the Source data. Additional supporting data are available from the corresponding authors upon reasonable request (response within two weeks). All requests for raw and analyzed data and materials will be reviewed by the corresponding authors to verify if the request is subject to any intellectual property or confidentiality obligations. Donor-related data not included in the paper were generated as part of clinical examination and may be subject to patients confidentiality. Any data and materials that can be shared will be released via a Material Transfer Agreement.
Code availability: R code to reproduce the analyses of multiparametric flow-cytometry data is available online: https://github.com/teximmed2-fr/flu-specific-CD4-T-cells. The R code is citable with following DOI: 10.5281/zenodo.5541093.

Field-specific reporting

Please select the one below that is the best fit for your research. If you are not sure, read the appropriate sections before making your selection.

☒ Life sciences ☐ Behavioural & social sciences ☐ Ecological, evolutionary & environmental sciences

For a reference copy of the document with all sections, see nature.com/documents/nr-reporting-summary-flat.pdf

Life sciences study design

All studies must disclose on these points even when the disclosure is negative.

Sample size	22 volunteer healthy donors were recruited and were vaccinated with seasonal influenza vaccines in the time periods from 2016 until 2020. Donor material was banked at the University Hospital Freiburg; (1) Antigen-specific CD4 T cell analysis was performed with samples from 12 DRB1*01:01-positive vaccinated individuals. (2) Vaccine-specific and HA-specific IgG levels were analysed in all 22 donors. No sample size calculations were performed. 22 volunteer donors gave informed consent and were available to donate blood sample. The sample number is comparable to many other studies in the research field of human immunology and proved to be sufficient for the generation of reproducible results- (Citations: doi: 10.1016/j.jhep.2019.02.016, doi: 10.1172/JCI129642)
Data exclusions	For flow cytometrical analysis, cell populations containing less than 5 cells were excluded. This data exclusion strategy has been applied and validated previously by our group to gain reproducible results in studies investigating virus-specific CD4+ T cells in human viral infections.
Replication	Analyses were performed in independent experiments. Independent experiments: flow cytometry analysis: for analysis including panel 1 and 2: 21 (HA118-132), for analysis with panel 1: 11 (HA118-132), for analysis with panel 2: 11(HA118-132), and 6 (HA306-318) and plasma analysis: 22 donors ,independent of HLA type.<br>Findings were reproducible: For example: Panel 1 and Panel 2 include same markers like: ICOS, CD38, CXCR5, CXCR3, PD-1 and so on: Longitudinal analysis of #1-2019 with both panels (1+2) showed the same results. Otherwise human samples are very limited, so it is not possible to perform experiments repeatedly.
Randomization	Donors were selected based on availability and HLA-typing (DRB1*0101). The covariates age and gender are well-documented at Table 1.
Blinding	Blinding was not applied. Non-objective parameters were not included in the study design. Due to standardized analyses of the flow cytometric data set, biased analysis can be excluded.

Reporting for specific materials, systems and methods

We require information from authors about some types of materials, experimental systems and methods used in many studies. Here, indicate whether each material, system or method listed is relevant to your study. If you are not sure if a list item applies to your research, read the appropriate section before selecting a response.

Materials & experimental systems

n/a	Involved in the study	
☐	☒	Antibodies
☒	☐	Eukaryotic cell lines
☒	☐	Palaeontology and archaeology
☒	☐	Animals and other organisms
☐	☒	Human research participants
☒	☐	Clinical data
☒	☐	Dual use research of concern

Methods

n/a	Involved in the study	
☒	☐	ChIP-seq
☐	☒	Flow cytometry
☒	☐	MRI-based neuroimaging

Antibodies

Antibodies used

Flow Cytometry:

BD Biosciences

anti-CD4-FITC (RPA-T4, 8037703, 1:50) Cat:#555346
anti-CCR7-BUV395 (3D12, 43026, 4:100) Cat:#563977
anti-CXCR5-APC (RF8B2, 558113, 1:50) Cat:#356908
anti-PD-1-BV786 (EH12.1, 1013122, 3:100) Cat:#563789
anti-CD134(OX40)-PE-Cy7 (ACT-35, 65008, 3:100) Cat:#563663
anti-CD38-BUV737 (HB7, 8339566, 1:200) Cat:#564686
anti-ICOS-BV711 (DX29, 7164540, 1:100) Cat:#563833
anti-CD127-BV421 (HIL-7R-M21, 9346341, 3:100) Cat:#562436
anti-CCR6-BV605 (11A9, 9065844, 3:100) Cat:#562724
anti-T-bet-PE-CF594 (O4-46,93533305, 3:100) Cat:#562467

Biolegend

anti-CD4-AlexaFluor700 (RPA-T4, 300526, 1:200) Cat:#300526
anti-CXCR5-BV421 (J252D4, B252332, 1:100) Cat:#562747
anti-CXCR3-BV510 (G025H7, B293007, 3:100) Cat:#353726
anti-CD127-BV605 (A019D5, B320426, 3:100) Cat:#351334
anti-CD27-PE-Dazzle594 (M-T271, B291287, 1:400) Cat:#356422
anti-Ki67-PE-Cy7 (Ki67, B269861, 1:200) Cat:#350525

Cell signaling

anti-TCF-1-AlexaFluor488 (C63D9, 11, 1:100) Cat:#6444

eBioscience

anti-CD14-eFluor780 (61D3, 2152036, 1:100) Cat:#47-0149-42
anti-CD19-eFluor780 (HIB19, 2145095, 1:100) Cat:#47-0199
anti-ViaDye-eFluor780 (1968321, 1:200) Cat:#65-0865
anti-TOX-eFluor660 (TRX10, 1995394, 1:100) Cat:#50-6502

Invitrogen

anti-CD45RA-PerCP-Cy5.5 (HI100, 1993639, 3:100) Cat:#45-0458-42
2-NBDG (20mM) (1:200) Cat:#N13195

Sigma-Aldrich

anti-human IgG (HRP) (polyclonal, 1:3000) Cat: A0293-1ML

Jackson ImmunoResearch

anti-human Fcgamma F(ab')2 fragment (Horseradish Peroxidase) (polyclonal, 1:20000) Cat: 109-036-008

Validation

All antibodies were obtained from commercial cendors and we based specificity on descriptions and information provided in corresponding data sheets available and provided by the manufacturers. Standardized analysis in different cohorts, antibody titration on PBMCs including unstained controls, comparisons of different antibody clones and conjugates and validated by publications:

CD4, clone RPA-T4 : antibody titration on PBMCs; control clones SK3; using B cells as negative control
CCR7, clone 3D12 : antibody titration on PBMCs; control clone G043H7; validated with respect to differential expression of naïve and non-naïve T cell subpopulations
CXCR5, clone RF8B2 and J252D4: antibody titration on PBMCs; control clone MU5UBEE; validated with respect to differential expression of naïve and non-naïve T cell subpopulations
PD-1, clone EH12.2H7: antibody titration on PBMCs; control clones eBioJ105; validated with respect to differential expression of naïve and non-naïve T cell subpopulations
CD134, clone ACT-35: antibody titration on PBMCs; control clones L106; validated with respect to differential expression of naïve and non-naïve T cell subpopulations
CD38, clone HB7: antibody titration on PBMCs; control clone HIT2; validated with respect to differential expression of naïve and non-naïve T cell subpopulations
ICOS, clone DX29: antibody titration on PBMCs; control clone ISA-3; validated with respect to differential expression of naïve and non-naïve T cell subpopulations
CD127, clone HIL-7R-M21 and A019D5: antibody titration on PBMCs; control clone eBioRDR5; validated with respect to differential

expression of naïve and non-naïve T cell subpopulations
CCR6, clone 11A9: antibody titration on PBMCs; validated with respect to differential expression of naïve and non-naïve T cell subpopulations
T-bet, clone O4-46: antibody titration on PBMCs; control clones 4B10; validated with respect to differential expression of naïve and non-naïve T cell subpopulations
CXCR3, clone G025H7: antibody titration on PBMCs; control clone 1C6/CXCR3; validated with respect to differential expression of activated and non-activated T cell subpopulations
CD27, clone M-T271: antibody titration on PBMCs; control clone O323; validated with respect to differential expression of naïve and non-naïve T cell subpopulations
Ki67, clone Ki67: antibody titration on PBMCs; control clone B56; validated with respect to differential expression of naïve and non-naïve T cell subpopulations
TCF-1, clone C63D9: antibody titration on PBMCs; control clone 7F11A10; validated with respect to differential expression of naïve and non-naïve T cell subpopulations
CD14 , clone 61D3: antibody titration on PBMCs; control clones M5E2 and MϕP9; using T cell populations as negative control
CD19, clone HIB19: antibody titration on PBMCs; control clone SJ25C1; using T cell populations as negative control
Viability Dye was titrated on PBMCs; validated with respect to differential staining of live and dead cell populations
TOX1, clone TRX10: antibody titration on PBMCs; control clone REA473; validated with respect to differential expression of naïve and non-naïve T cell subpopulations
CD45RA, clone HI100: antibody titration on PBMCs; validated with respect to differential expression of naïve and non-naïve T cell subpopulations
2-NBDG: antibody titration on PBMCs; validated with respect to differential expression of naïve and non-naïve T cell subpopulations

Human research participants

Policy information about studies involving human research participants

Population characteristics

22 healthy donors were recruited at the University Hospital Freiburg. 12 of 22 donors carry the HLA-DRB101*01 while 10 of 22 donors have another HLA-type. 10 of 22 donors participated more than one time. The gender ratio of donors was m/f: 8/14. At study entry of the respective subjects, the median age was 26.9 years.

Recruitment

Healthy donors were recruited at the University Hospital Freiburg. Self-selection bias or other biases can be excluded since several people were included in the recruitment. Samples were banked and retrospectively selected according to the following inclusion criteria: HLA-DRB1*0101. Additional donors independent on HLA typing were used to measure influenza-specific IgG (vaccine and HA-specific IgG).

Ethics oversight

Written informed consent was obtained from all participants and the study was conducted according to federal guidelines, local ethics committee regulations (Albert-Ludwigs-Universität, Freiburg, Germany; vote #: 322/20) and the Declaration of Helsinki (1975).

Note that full information on the approval of the study protocol must also be provided in the manuscript.

Flow Cytometry

Plots

Confirm that:

☒ The axis labels state the marker and fluorochrome used (e.g. CD4-FITC).

☒ The axis scales are clearly visible. Include numbers along axes only for bottom left plot of group (a 'group' is an analysis of identical markers).

☒ All plots are contour plots with outliers or pseudocolor plots.

☒ A numerical value for number of cells or percentage (with statistics) is provided.

Methodology

Sample preparation

Cryopreserved isolated human PBMCs were thawed and prepared for flow cytometry decribed in the methods section. Plasma was thawed for vaccine-specific and HA-specific IgG ELISA.

Instrument

LSRFortessa (BD, Germany) Spark reader (TECAN GmbH, Germany) and BioTek (Winooski, VT, USA)

Software

FlowJo_v10.0.7 (LLC, BD Life Sciences, Ashland, OR, USA), R version 3.6.3 using the ComplexHeatmap package (Gu ZG, Eils R, Schlesner M (2016)), Omiq (Omiq, Inc.), SparkControl magellan software 2.2.

Cell population abundance

Abundance of influenza-specific CD4 T cells is low (>10^-3 %)

Gating strategy

Lymphocytes gated on FSC-A and SSC-A, Doublet exclusion on FSC-H and FSC-W and SSC-H and SSC-W, Exclusion of dead cells, B cells and monocytes, Gating on CD4 T cells, Exclusion of naive cells (CCR7+CD45RA+), Gating of influenza-specific CD4 T cells via tetramers are shown in the Supplementary Fig. 11.

☒ Tick this box to confirm that a figure exemplifying the gating strategy is provided in the Supplementary Information.
